# Hitting the Target: Model-Informed Precision Dosing of Tobramycin in Pediatric Patients with Cystic Fibrosis

**DOI:** 10.3390/ph19010150

**Published:** 2026-01-14

**Authors:** Jake M. Brockmeyer, Laura Bio, Carlos Milla, Adam Frymoyer

**Affiliations:** 1 Department of Pharmacy, Lucile Packard Children’s Hospital Stanford, Palo Alto, CA 94304, USA; 2 Division of Pediatric Pulmonary Medicine, Stanford University, Stanford, CA 94305, USA; 3 Department of Pediatrics, Stanford University, Stanford, CA 94305, USA

**Keywords:** model-informed precision dosing, cystic fibrosis, tobramycin, pediatrics, pharmacokinetics, therapeutic drug monitoring, area under the curve, clinical decision support

## Abstract

**Background:** Tobramycin is a key therapy for pulmonary exacerbations in children and adolescents with cystic fibrosis (CF), yet its variable pharmacokinetics (PK) combined with narrow therapeutic index necessitates therapeutic drug monitoring (TDM) during clinical care to optimize exposure while minimizing toxicity. Model-informed precision dosing (MIPD) is a potentially powerful tool to support dose individualization in clinical care that leverages population PK models and Bayesian forecasting. Herein, we evaluated the performance of an MIPD initiative at our hospital for once-daily tobramycin in pediatric patients with CF. **Methods:** Tobramycin practices at a single CF center before (2016–2018) and after (2019–2025) implementation of an MIPD initiative in CF patients < 21 years were evaluated. TDM during the pre-MIPD period used traditional log-linear AUC calculations, while the post-MIPD period used a commercial MIPD software platform integrated within the electronic health record. Outcomes included attainment of the target 24 h area-under-the-curve (AUC_24_ 80–120 mg·h/L), number of TDM samples and dose adjustments during the first 7 days of treatment, and rates of acute kidney injury (AKI). **Results:** A total of 114 treatment courses were analyzed (77 pre-MIPD, 37 post-MIPD). Post-MIPD target attainment was 75.7% at TDM1, 89.2% at TDM2, and 100% at TDM3, significantly higher than pre-MIPD at corresponding cycles. The post-MIPD period required fewer TDM samples (4.2 vs. 7.1; *p* < 0.001) and dose adjustments (0.7 vs. 1.8; *p* < 0.001) in the first 7 days. AKI incidence remained low and comparable between periods. **Conclusions:** Implementation of an MIPD initiative for tobramycin in pediatric patients with CF led to the early attainment of therapeutic AUC_24_ targets while reducing TDM burden and dose adjustments.

## 1. Introduction

Pulmonary exacerbations in children and adolescent patients with cystic fibrosis (CF) are a major driver of morbidity. Tobramycin remains a cornerstone for anti-pseudomonal coverage during pulmonary exacerbations and extended-interval (once-daily) dosing is widely adopted to leverage aminoglycosides’ concentration-dependent killing and post-antibiotic effect while mitigating nephrotoxicity [1,2,3,4]. Yet, the pharmacokinetics (PK) of tobramycin in pediatric CF are highly variable due to age-related physiology and altered body composition [5,6,7,8]. As a result, therapeutic drug monitoring (TDM) is essential to ensure adequate exposure for efficacy while minimizing toxicity. Increasingly, tobramycin exposure optimization has focused on the area under the concentration–time curve over 24 h (AUC_24_) as this pharmacokinetic/pharmacodynamic target has been demonstrated to be a predictor of bacteria killing, clinical efficacy, and acute kidney injury (AKI) [9,10,11,12,13].

AUC-guided dosing can be difficult to implement in clinical care. It requires multiple timed concentrations, complex PK calculations, and potentially additional training for clinicians and pharmacists. In addition, traditional AUC calculations based on simple first-order PK equations require precise sample timing, assumption of a one-compartment model, and steady-state kinetics [14], all of which may not be valid during clinical care. Our previous work characterized the challenges of implementing AUC-guided dosing using traditional approaches for tobramycin in a pediatric CF population and demonstrated low target attainment, need for repeated TDM sampling, and inconsistent practice [15].

Model-informed precision dosing (MIPD) offers a powerful solution to support AUC-guided dosing [16,17]. MIPD leverages Bayesian forecasting anchored to high-quality population PK models and patient information (e.g., demographics, renal function, dosing history, infusion times, serum concentrations) to estimate a patient’s individual PK and exposure. The approach can readily handle the sparse, highly variable data that are frequently encountered in clinical care, and MIPD does not require steady-state assumptions. User-friendly software platforms supporting MIPD have also been developed that can be readily integrated within the electronic health record (EHR) offering point of care accessibility and reduced burden of manual data entry [18,19]. Taken together, MIPD offers the potential to support the timely calculation of AUC, evaluation of dosing regimens, and development of personalized dose recommendations tailored to each patient in a standardized way across providers.

We previously implemented an AUC-guided MIPD initiative in clinical care for vancomycin in pediatric patients with CF at our hospital, demonstrating the reliable delivery of Bayesian dosing in routine care and improved target attainment [20]. Building on that experience, we developed a CF-specific MIPD initiative for once-daily tobramycin and implemented it in clinical care in December 2019 as part of a quality improvement initiative [15]. The present evaluation describes the implementation of this initiative and assesses its clinical performance in routine care, including exposure target attainment, TDM burden, and safety outcomes.

## 2. Results

### 2.1. Patients

This study was an evaluation of a tobramycin MIPD initiative implemented in patients with CF at a university-affiliated free-standing children’s hospital that is a Cystic Fibrosis Foundation accredited CF center. Two time periods were evaluated: pre- and post-implementation of the MIPD initiative. A total of 114 treatment courses were available for evaluation, with 77 treatment courses in the pre-MIPD period and 37 treatment courses in the post-MIPD period. Characteristics of patients at each treatment course during both periods were similar (Table 1) except the duration of tobramycin treatment trended toward shorter courses in the post-MIPD vs. pre-MIPD period (pre-MIPD 13.5 ± 6.2 days vs. post-MIPD 11.3 ± 3.6, *p* = 0.051). This finding aligns with general practice changes toward shorter antibiotics courses for patients with CF [21]. Concomitant antipseudomonal antibiotic use also differed between periods for piperacillin/tazobactam (pre-MIPD 68.8% vs. post-MIPD 24.0%, *p* < 0.001) and cefepime (pre-MIPD 15.6% vs. post-MIPD 43.0%, *p* = 0.002). Ceftazidime, meropenem, and fluoroquinolone concomitant therapy were not statistically different between periods. The number of concomitant nephrotoxic medications patients was comparable between periods.

### 2.2. Outcomes

**Figure 1:**
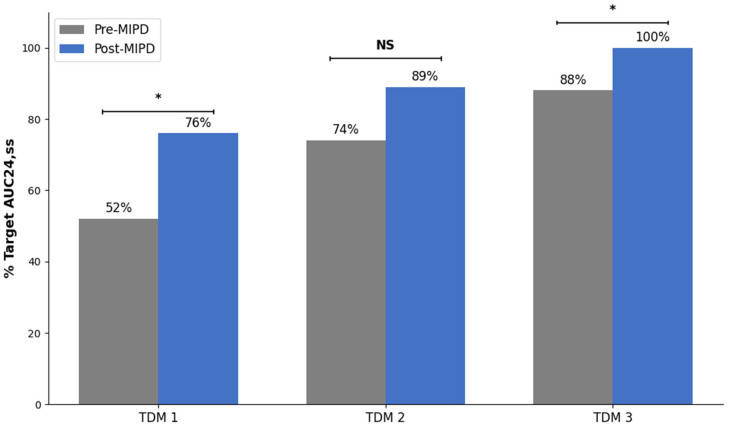
Achievement of tobramycin target AUC_24,ss_ 80–120 mg·h/L by therapeutic drug monitoring (TDM) cycle pre- and post-implementation of a model-informed precision dosing (MIPD) initiative. Pre-MIPD represents n = 77 treatment courses. Post-MIPD represents n = 37 treatment courses. * denotes *p* < 0.05 pre-MIPD vs. post-MIPD via Fisher’s exact test. NS, not significant at *p* < 0.05. AUC_24,ss_ area under the curve over 24 h at steady state.

During the post-MIPD period, achievement of the target steady-state 24 h AUC 80–120 mg·h/L was 75.7% (28/37) at TDM 1 (which reflects the starting dose recommendation). Target achievement improved to 89.2% (33/37) at TDM 2, and all patients (37/37) were at target by TDM 3. In comparison, applying the same AUC_24,ss_ target to the pre-MIPD period (Figure 1), achievement was lower than during the post-MIPD period at TDM 1 (*p* < 0.05) and TDM 3 (< 0.05) with a nonsignificant trend toward lower target achievement at TDM 2 (*p* = 0.086).

**Figure 2:**
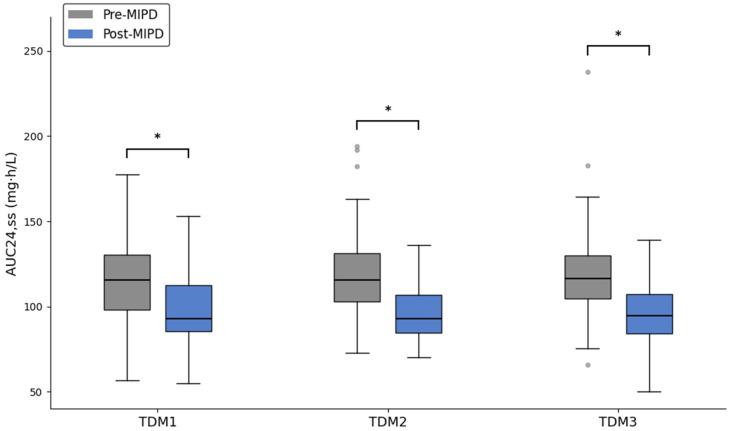
Boxplots of tobramycin AUC_24,ss_ (mg·h/L) by therapeutic drug monitoring (TDM) cycle pre- and post-implementation of a model-informed precision dosing (MIPD) initiative. Boxes show the median (line), 25th–75th percentiles (box), and data within 1.5 × interquartile range (whiskers); outliers are shown as circles. Pre-MIPD represents n = 77 treatment courses. Post-MIPD represents n = 37 treatment courses. * denotes *p* < 0.001 pre-MIPD vs. post-MIPD via Mann–Whitney U.

AUC_24,ss_ at each TDM cycle by period is shown in Figure 2. The median AUC_24,ss_ was lower in the post-MIPD period compared to the pre-MIPD period at all TDM cycles (all *p* ≤ 0.001). The spread of the interquartile range was narrower post-implementation of MIPD, reflecting a decreased variation in exposure between patients.

The starting dose was on average lower in the post-MIPD period compared to the pre-MIPD period (pre-MIPD 12.7 ± 2.3 mg/kg/day vs. post-MIPD vs. 11.1 ± 2.0 mg/kg/day; *p* = 0.001). When stratifying by age group for the post-MIPD period, the mean starting dose was 12.0 ± 2.2 for ≤12 years and 11.0 ± 1.8 mg/kg for >12 years. For the pre-MIPD period, the mean starting dose was 12.6 ± 2.5 for ≤12 years and 12.5 ± 2.2 mg/kg for >12 years.

**Figure 3:**
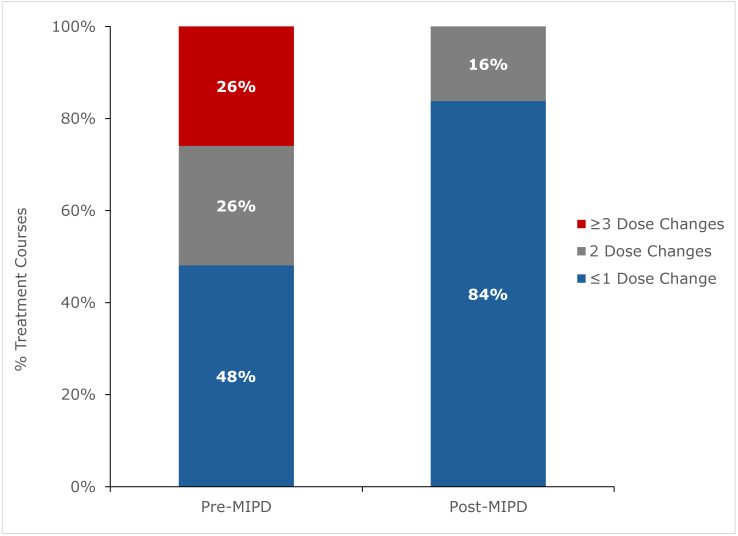
Distribution of the number of dose changes in patients during the first 7 days of treatment pre- and post-implementation of a model-informed precision dosing (MIPD) initiative. Pre-MIPD represents n = 77 treatment courses. Post-MIPD represents n = 37 treatment courses. The distribution differed between periods at *p* < 0.001 via Fisher’s exact test.

The number of TDM cycles during the first 7 days of treatment was similar between periods (pre-MIPD 3.3 ± 1.4 vs. post-MIPD 2.9 ± 1.0; *p* = 0.156). However, the number of serum concentrations measured as part of TDM was lower in the post-MIPD period compared to the pre-MIPD period (pre-MIPD 7.1 ± 3.2 vs. post-MIPD 4.2 ± 1.2; *p* < 0.001). Additionally, the number of dose adjustments during the first 7 days of treatment was lower in the post-MIPD period (pre-MIPD mean 1.8 ± 1.4 vs. post-MIPD mean 0.7 ± 0.7, *p* < 0.001). Figure 3 shows the distribution of the number of dose changes during the first 7 days of treatment. Notably, 52% (40/77) of treatment courses required two or more dose changes during the first 7 days of treatment in the pre-MIPD period vs. only 16% (6/37) of treatment courses during the post-MIPD period (*p* < 0.001).

The incidence of AKI as detected by serum creatinine [SCr] was low and did not differ between periods (pre-MIPD 7.8% [6/77] vs. post-MIPD 5.4% [2/37]; *p* = 1.0). Of the eight patients who developed AKI, seven were on at least one other concomitant nephrotoxic medication. One patient in the post-MIPD period developed stage 3 oliguric AKI on day 12 of treatment requiring intermittent hemodialysis. His dose was stable throughout treatment (8.9 mg/kg) and his AUC_24,ss_ was within the target at TDM 1–3 (103–114 mg·h/L). This patient discontinued intermittent hemodialysis prior to discharge and SCr returned to baseline shortly thereafter. Notably, the patient was exposed to one concomitant nephrotoxic medication (piperacillin/tazobactam) and received tobramycin for 8 days one month prior to this treatment course.

## 3. Discussion

We implemented an MIPD initiative for tobramycin in pediatric patients with CF that was supported by a cloud-based, EHR-integrated CDS tool enabling AUC-guided dosing. With MIPD, 89% of patients achieved the target AUC_24,ss_ of 80–120 mg·h/L after a single TDM cycle with dose adjustment, and 100% were at target after the second cycle. In the pre-MIPD period, 74% and 88% of patients achieved the target AUC_24,ss_ after the first and second TDM cycle, respectively. The overall TDM burden in the first 7 days of treatment was also lower in the post-MIPD period than in the pre-MIPD period, with fewer drug concentration measurements and fewer dose adjustments. These findings are consistent with our prior experience implementing an MIPD program to guide vancomycin dosing in pediatric patients with CF [20,22].

MIPD offers a solution to the challenge of optimizing tobramycin therapy in CF care, where balancing effective drug exposure while minimizing toxicity is complicated by disease-specific pathophysiology and clinical complexity [1,9,23]. Patients with CF often exhibit substantial variability in the antimicrobial volume of distribution and clearance, including episodes of augmented renal clearance during pulmonary exacerbations [24,25,26,27,28]. Consequently, TDM with dose optimization is essential, even when empiric regimens are carefully calibrated at the population level. MIPD provides a robust, standardized framework to estimate individual PK parameters from patient-specific characteristics and sparse, flexibly timed (non-trough-centric) samples. This patient-specific understanding enables personalized dosing and a more consistent attainment of exposure targets. Our experience highlights the real-world clinical use of MIPD in pediatric CF and demonstrates high performance in achieving target exposures. Moreover, MIPD can be readily recalibrated as the clinical status evolves (e.g., changes in kidney function), supporting the maintenance of target exposure throughout therapy.

As part of our MIPD initiative, we applied a Bayesian modeling and simulation framework using our own local data to update the institutional starting dose to 12 mg/kg IV every 24 h for patients < 12 years and 10 mg/kg for those ≥12 years. This empiric, age-stratified strategy improved initial target attainment in the post-MIPD period (76% at TDM1). However, persistent between-patient variability limited the effectiveness of fixed starting doses, further highlighting the need for individualized dosing. The Bayesian MIPD workflow remained essential for reliably achieving target exposures, as evidenced by the rapid convergence to goal (89% at TDM2 and 100% at TDM3).

Beyond improved exposure target attainment, MIPD implementation yielded additional benefits. The reduced TDM burden is particularly valuable in pediatrics, where frequent blood draws impose substantial stress on patients and families. We observed a mean reduction of approximately three serum concentration measurements during the first 7 days of therapy, representing a clinically meaningful decrease in blood volume and painful phlebotomy events. Corresponding reductions in laboratory costs and phlebotomy/nursing workload are additional potential advantages.

Aminoglycoside-associated nephrotoxicity is a recognized concern in pediatric CF, influenced by prior aminoglycoside exposure, longer treatment duration, and concomitant nephrotoxins [29]. At our hospital, the incidence of AKI was low and similar across evaluation periods (pre-MIPD 7.8% vs. post-MIPD 5.4%), with most events occurring alongside additional nephrotoxic agents. Our use of a modified KDIGO definition incorporating an absolute serum creatinine threshold (≥0.5 mg/dL) may under-detect very mild creatinine elevations in children with low baseline values. This approach was intentionally chosen to reduce misclassification of clinically insignificant fluctuations labeled as AKI and was consistent with nephrotoxicity stewardship practices [30]. The single severe AKI case in the post-MIPD period followed a recent prior tobramycin course despite on-target AUCs during the index treatment course, underscoring the importance of considering cumulative aminoglycoside exposure over time [31,32]. These observations suggest that while MIPD may help avoid unnecessary exposure during individual courses, ongoing nephrotoxicity stewardship, minimizing concomitant nephrotoxins and tracking cumulative aminoglycoside exposure, remains essential to further reduce AKI risk in pediatric patients with CF.

Several limitations should be acknowledged. Our data represent the experience of an MIPD initiative at a single center and therefore might limit generalizability. The retrospective design, along with the differences in dosing methods, sampling schedules, and exposure targets across the two periods, moderates direct comparisons between the periods and could overestimate the benefits attributable solely to MIPD. We applied the same exposure target (AUC_24,ss_ 80–120 mg·h/L) to both periods, as this target reflects what was used during clinical care in the post-MIPD period, which was the primary focus of the current project. During the pre-MIPD period, a narrower exposure range (AUC_24,ss_ 100–125 mg·h/L) was used in clinical care. Had this historical target been used for evaluation, target attainment in the pre-MIPD period would have been lower (TDM1 27%, TDM2 45%, TDM3 64%). Irrespective of direct between-period comparisons, our project clearly demonstrates the performance of an MIPD initiative in a real-world clinical setting.

An additional limitation is the relatively small sample size (n = 37 treatment courses) post-MIPD reflecting contemporary CF care characterized by fewer hospitalizations for pulmonary exacerbations due to widespread CFTR modulator use, evolving treatment practices, and the COVID-19 pandemic. Consequently, patients requiring inpatient tobramycin in the post-MIPD period may represent a more selected population with greater disease severity or refractory exacerbations, limiting direct comparison with the pre-MIPD cohort. In addition, the modest post-MIPD sample size reduces statistical power, particularly for detecting small between-period differences and evaluating infrequent outcomes such as AKI. As such, comparisons between periods should be interpreted with caution. Nevertheless, the consistency and magnitude of improvements observed across multiple process-of-care metrics support the robustness of our implementation findings in a contemporary CF care landscape.

Lastly, barriers to MIPD implementation are present including costs for health care personnel training, information technology support, and software licenses [18,33]. Successful adoption also requires collaboration among hospital stakeholders and the identification of local MIPD champions [18,33]. Although the cost-effectiveness of tobramycin MIPD has not yet been established, cost–benefit analyses of vancomycin have demonstrated savings with MIPD compared with conventional TDM even after accounting for commercial software licensing costs driven by reductions in TDM intensity, laboratory testing, and AKI [34,35]. Further research evaluating the cost-effectiveness and impacts on clinical outcomes will help facilitate broader MIPD implementation at the bedside.

## 4. Methods

### 4.1. Patient Population

Patients with CF were included if they were <21 years, treated with once-daily intravenous tobramycin for ≥7 days, and had at least two measured tobramycin serum concentrations. Tobramycin, in combination with an antipseudomonal beta lactam antibiotic, is first-line treatment at our hospital for patients with CF who have a pulmonary exacerbation and history of *Pseudomonas aeruginosa* based on respiratory culture. Patients were excluded if they had undergone a lung transplant or required extracorporeal membrane oxygenation during tobramycin treatment. More than one treatment course for a patient was included in the analysis if separated by ≥14 days between courses.

### 4.2. Pre-MIPD Period

In the pre-MIPD period (January 2016 to February 2018), a tobramycin starting dose of 12–15 mg/kg IV every 24 h was recommended. TDM was performed after the first or second dose to evaluate exposure and individualize dosing. As part of TDM, two timed tobramycin drug concentrations were collected at 2 and 8 h after the start of the infusion. Steady state was assumed and AUC_24,ss_ was calculated using standard log-linear regression equations embedded within an Excel spreadsheet (Microsoft, Redmond, WA, USA) template [14]. Doses were adjusted to achieve an AUC_24,ss_ 100–125 mg h/L. TDM and dose adjustments were managed per the physician and clinical pharmacist involved with the patient’s treatment team. No formal guidelines for tobramycin management in patients with CF were in place.

### 4.3. Post-MIPD Period

Through a multidisciplinary team of physicians and pharmacists, a formal MIPD initiative for once-daily tobramycin in pediatric patients with CF was developed and approved by the hospital’s Pharmacy and Therapeutics Committee in November 2019. As part of the initiative, a formal institutional “Guideline for Tobramycin in Cystic Fibrosis Patients” was also developed (see Figure S1). The initiative went live in clinical care in December 2019.

A detailed description of MIPD implementation at our hospital has been previously published [18]. In short, MIPD was implemented within a commercial, cloud-based CDS tool integrated within the EHR (InsightRx^®^, San Francisco, CA, USA) [18]. The MIPD CDS tool has been used to support vancomycin MIPD at our hospital since 2017. The same platform and processes were applied to support tobramycin MIPD. Relevant patient data were transmitted directly from the EHR via secure application programming interface (API) calls, minimizing manual data entry and transcription errors. The MIPD CDS tool also incorporates automated data validation and error-detection procedures to support data quality and dosing accuracy. The system automatically screens for implausible or inconsistent data, including invalid dosing or laboratory units, duplicate or temporally implausible dose administrations, drug concentrations obtained too close to infusion times, extreme values, and missing covariates required for PK calculations. When such discrepancies are detected, users are alerted through warning or error notifications, and PK calculations are paused until the data are corrected or excluded.

Clinical pharmacists underwent training on tobramycin therapeutic principles for CF and use of the MIPD clinical decision support (CDS) tool. Training consisted of short staff-wide in-service sessions with pre and post competency assessments to ensure proficiency. An MIPD oversight committee met for operational reviews and process optimization. This included regular stakeholder communications and structured feedback to end users.

The MIPD CDS tool used a maximum a priori (MAP) estimation and Bayesian forecasting method integrating a patient’s dosing history, drug concentrations, and patient characteristics (i.e., fat-free mass, age, SCr) to estimate a patient’s individual PK, exposure, and dose needs. For tobramycin, the Hennig et al. population PK model developed in 465 children and adults with CF and 267 children and adults without CF served as the a priori [8]. In brief, it is a two-compartment model with first-order elimination. Clearance is predicted by fat-free mass, age, and SCr standardized for age. Central volume, peripheral volume, and inter-compartment clearance are predicted by fat-free mass. Exponential or proportional error models capture between-patient, within-patient, and between-occasion variability. We previously demonstrated the predictive performance of the Hennig et al. model in our population of pediatric patients with CF [15].

During the post-MIPD period (December 2019 to July 2025), the target AUC_24,ss_ was 80–120 mg h/L. This target was chosen based on pharmacodynamic considerations including clinical studies in tobramycin in patients with CF with pulmonary exacerbations that showed an AUC_24_/MIC > 40 to 50 which was associated with improved clinical outcomes [8,10,13]. At an MIC of ≤2 mg/L, an AUC_24,ss_ 80–120 mg h/L will routinely achieve this AUC_24_/MIC target. AUC_24_ 80–120 mg h/L is also commonly used at other CF centers in the United States [36].

The recommended starting dose was 12 mg/kg IV every 24 h for <12 years and 10 mg/kg every 24 h for ≥12 years. This starting dose strategy was supported by a population PK modeling and simulation analysis of patients from the pre-MIPD period using the Hennig et al. population PK model [8]. Doses of 10–16 mg/kg IV every 24 h were examined. The post-MIPD period starting dose strategy was predicted to achieve an AUC_24,ss_ 80–120 mg*h/L in 66% of <12 year olds and 69% of ≥12 year olds.

In the post-MIPD period, TDM was performed after the first dose and supported by MIPD. At this first TDM cycle, two tobramycin concentrations were recommended with flexible sampling times of 1.5 to 3.5 h and 6 to 10 h after start of infusion. The flexible sampling windows facilitated integration of TDM with routine clinical workflows including coordinating with other clinical labs. The sampling windows were supported by two prior evaluations of the Hennig et al. population PK model, which demonstrated similar predictive performance of Bayesian AUC estimation across multiple two-sample timing combinations spanning early and late post-infusion windows, including those used in the present study [37,38]. Utilizing the two tobramycin concentrations, AUC_24,ss_ was calculated within the MIPD CDS tool and doses were adjusted to achieve the target AUC_24,ss_ 80–120 mg h/L. Once at therapeutic target, subsequent TDM was recommended every 3 to 5 days. A single ‘random’ tobramycin concentration was permitted for subsequent TDM if the patient was stable and the MIPD CDS tool indicated a good model fit.

During both periods, tobramycin concentrations were quantified using a homogeneous enzyme immunoassay (Roche Diagnostic Corp, Indianapolis, IN, USA). The reportable range of the assay was 0.33–10 mg/L with samples diluted and reassayed as needed. The within-run and between-run coefficients of variation were less than 5%.

### 4.4. Study Data and Outcomes

Data were obtained retrospectively from the electronic health record as part of routine clinical care and included patient demographics, drug dosing information, tobramycin serum concentrations, serum creatinine levels, concomitant medications, and clinical course. The primary outcome was attainment of the target AUC_24,ss_ 80–120 mg·h/L. This exposure target was selected as it reflects the pharmacokinetic/pharmacodynamic and clinical standards in clinical practice during the post-MIPD period, which was the primary focus of our evaluation. To allow a consistent assessment of exposure attainment across the evaluation periods, this same target AUC_24,ss_ was applied to both the pre- and post-MIPD cohorts.

At each TDM cycle, the rate of target AUC_24,ss_ 80–120 mg·h/L achievement at each was evaluated. A TDM cycle entailed up to three (peak, random, and/or trough) measured serum concentrations, estimation of AUC_24,ss_, and dose adjustment as clinically needed. The first three TDM cycles during a treatment course were included for analysis. During the post-MIPD period, AUC_24,ss_ was calculated prospectively using the MIPD CDS tool. For the pre-MIPD period, AUC_24,ss_ was calculated retrospectively using the nonlinear mixed-effects modeling software NONMEM 7.3 (ICON Development Solutions, Ellicott City, MD, USA), and the same MAP estimation and Bayesian forecasting methodology employed as during the post-MIPD period was used.

Secondary outcomes included the starting dose, the number of dose adjustments and the number of TDMs performed during the first 7 days of therapy, and the duration of treatment. The incidence of AKI was evaluated using a modified KDIGO definition approach defined as a ≥1.5-fold increase in SCr from baseline, provided the absolute SCr reached at least 0.5 mg/dL at any time during tobramycin treatment. This approach, consistent with recommendations from pediatric nephrotoxicity studies such as the Nephrotoxic Injury Negated by Just-in-Time Action (NINJA) group, was used to minimize overestimation of clinically significant AKI in children, who often have low baseline creatinine (<0.5 mg/dL) and negligible absolute SCr increases [30]. Concomitant nephrotoxin medications were collected as described by the NINJA group to understand additional AKI risk in patients [30].

### 4.5. Statistical Analysis

Differences between periods were compared with Student’s *t*-test or Mann–Whitney U for continuous data, and Fisher’s exact test for categorical variables. Continuous data are reported as mean ± standard deviation or median (interquartile range), and categorical data as n (%). Two-sided α was set at 0.05. Analyses were performed in Python 3.12.12 (SciPy) and STATA 13.1 (StataCorp LP).

## 5. Conclusions

The implementation of an MIPD initiative for once-daily tobramycin in pediatric patients with CF significantly improved the rate of achieving therapeutic AUC targets while reducing the burden of TDM. The transition to MIPD not only streamlined dose adjustments and minimized the number of required serum concentration measurements but also maintained a low incidence of AKI, underscoring its potential as a valuable tool in optimizing antibiotic therapy in this vulnerable population.

## Figures and Tables

**Table 1:** Characteristics of patients with cystic fibrosis at the start of each treatment course before and after implementation of a model-informed precision dosing (MIPD) initiative.

	Pre-MIPD (n = 77)	Post-MIPD (n = 37)	*p*-Value
Age, y	12.7 ± 5.0	13.5 ± 5.2	NS
BMI, kg/m^2^	18.4 ± 3.4	18.8 ± 3.8	NS
Female, n (%)	44 (57%)	20 (54%)	NS
Baseline eGFR ^a^, mL/min/1.73 m^2^	140 ± 55	145 ± 54	NS
Duration of therapy, days	13.5 ± 6.2	11 ± 3.5	NS
Concomitant antibiotics ^b^, n (%)			
Piperacillin/tazobactam	53 (69%)	9 (24%)	<0.001
Cefepime	12 (16%)	16 (43%)	0.002
Ceftazidime	9 (12%)	8 (22%)	NS
Meropenem	13 (17%)	3 (8%)	NS
Fluoroquinolone ^c^	21 (27%)	5 (14%)	NS
Number of concomitant nephrotoxic medications ^d^, n (%)			NS
0	18 (23%)	15 (41%)	
1	47 (61%)	17 (46%)	
2	11 (14%)	5 (14%)	
3	1 (1%)	0 (0%)	

All data are mean ± standard deviation or counts (%). NS, not significant at *p* < 0.05. ^a^ Estimated glomerular filtration rate (eGFR) using modified Schwartz equation; ^b^ Concomitant antipseudomonal antibiotics used during treatment course; ^c^ Ciprofloxacin, levofloxacin, or moxifloxacin; ^d^ Concomitant nephrotoxic medications as defined by NINJA study.

## Data Availability

The original contributions presented in this project are included in the article material. Further inquiries can be directed to the corresponding authors.

## Supplementary Materials

The following supporting information can be downloaded at https://www.mdpi.com/article/10.3390/ph19010150/s1, Figure S1: Guidelines for Tobramycin Dosing in Children with Cystic Fibrosis.

## Abbreviations

The following abbreviations are used in this manuscript:

AKI	acute kidney injury
AUC	area under the concentration-time curve
AUC_24_	area under the concentration-time curve over 24 h
AUC_24,ss_	area under the concentration-time curve over 24 h at steady state
BMI	body mass index
CDS	clinical decision support
CF	cystic fibrosis
CFTR	cystic fibrosis transmembrane conductance regulator
eGFR	estimated glomerular filtration rate
EHR	electronic health record
KDIGO	Kidney Disease: Improving Global Outcomes
MAP	maximum a posteriori
MIPD	model-informed precision dosing
NINJA	Nephrotoxic Injury Negated by Just-in-Time Action
PK	pharmacokinetics
SCr	serum creatinine
TDM	therapeutic drug monitoring
