# Supplementary material for: Hitting the Target: Model-Informed Precision Dosing of Tobramycin in Pediatric Patients with Cystic Fibrosis
Source: Pharmaceuticals (Basel). 2026 Jan 14;19(1):150. doi: 10.3390/ph19010150 (PMC12844645; doi:10.3390/ph19010150)
Supplement: Supplementary file 1 [file pharmaceuticals-19-00150-s001.zip › pharmaceuticals-4037580-supplementary.pdf]

Stanford Children's Health | Lucile Packard Children's Hospital Stanford

Guidelines for Tobramycin Dosing in Children with Cystic Fibrosis

Date: 11/2019
J. Brockmeyer, R. Wise, A. Frymoyer, L. Bio

INDICATIONS

Tobramycin is indicated for CF-related acute pulmonary exacerbation due to *Pseudomonas aeruginosa*

Duration of therapy: 10 to 21 days

INITIAL DOSING*

- Consider historical tobramycin courses and any corresponding therapeutic levels to guide
- Consider consult to inpatient pharmacy for model-based dosing using **Insight-Rx dosing platform**
- Standard tobramycin starting doses are listed in **Table 1**

Table 1. Tobramycin Starting Dose for Tobramycin Naive Patients

Patient	Initial Regimen
Age < 12 years	12 mg/kg IV q24h
Age ≥ 12 years	10 mg/kg IV q24h
eGFR <60 mL/min*	Consult inpatient pharmacy for model-based dose recommendations using **Insight-Rx dosing platform**

* If patient does not have documented serum creatinine in past 24 hours, a serum creatinine should be obtained.

THERAPEUTIC DRUG MONITORING (TDM)

When to measure first concentrations?

- After the first dose, obtain a "peak" (~2 hours after start of infusion) and "random" (~8 hours after start of infusion) tobramycin level
- If using Insight-Rx dosing platform timing of TDM levels can be flexible
  - Target within time range of 1.5-3.5 hours after start of infusion for 'peak' and 6 to 10 hours after start of infusion for "random"
  - Can time with clinical labs patient is scheduled

References

1. Arends A, Pettit R. Safety of Extended Interval Tobramycin in Cystic Fibrosis Patients Less Than 6 Years Old. J Pediatr Pharmacol Ther. 2018;23(2):152-158.
2. Gao Y, Hennig S, Barras M. Monitoring of Tobramycin Exposure: What is the Best Estimation Method and Sampling Time for Clinical Practice? Clin Pharmacokinet. 2019;58(3):389-399.
3. Barras MA, Serisier D, Hennig S, Jess K, Norris RLG. Bayesian Estimation of Tobramycin Exposure in Patients with Cystic Fibrosis. Antimicrob Agents Chemother. 2016;60(11):6698-6702.
4. Zobell JT, Epps K, Kittell F, et al. Tobramycin and Beta-Lactam Antibiotic Use in Cystic Fibrosis Exacerbations: A Pharmacist Approach. J Pediatr Pharmacol Ther. 2016;21(3):239-246.
5. Paviour S, Hennig S, Staatz CE. Usage and monitoring of intravenous tobramycin in cystic fibrosis in Australia and the UK. Journal of Pharmacy Practice and Research. 2016;46(1):15-21.
6. Mouton JW, Jacobs N, Tiddens H, Horrevorts AM. Pharmacodynamics of tobramycin in patients with cystic fibrosis. Diagnostic Microbiology and Infectious Disease. 2005;52(2):123-12700
7. Burkhardt O. Once-daily tobramycin in cystic fibrosis: better for clinical outcome than thrice-daily tobramycin but more resistance development? Journal of Antimicrobial Chemotherapy. 2006;58(4):822-829

GOAL TOBRAMYCIN EXPOSURE

- No well-established exposure target based on outcome data
- Tobramycin displays concentration dependent killing.
  - Area Under the Curve for 24 hours (AUC_{24}) or maximum concentration (C_{max})
- At LPCH, AUC_{24} is followed with target of **80 to 120 mg*h/L**
- AUC_{24} is estimated using the Insight-Rx dosing platform via model-based Bayesian forecasting.
  - If Insight-Rx not available two-point log-linear regression can be performed using standard PK equations (Excel calculators)

Table 2. Tobramycin Target Exposure

Indication	Suggested target	Other Considerations
CF-related acute pulmonary exacerbation due to *Pseudomonas aeruginosa*	AUC_{24} = 80 to 120 mg*h/L	C_{max} ≥ 20 mcg/ml may be an alternative or complementary exposure target for some patients.*

*C_{max} is defined as 1 hour after end of infusion

DOSE ADJUSTMENTs

- If patient not at target AUC_{24}, dose adjustment indicated.
- Use Insight-Rx dosing platform
  - Evaluate dosing regimens in platform by examining the predicted steady-state AUC_{24}
- What selecting dose and exposure target consider patient's evolving clinical status and changing renal function (i.e. acute kidney injury)
- Re-evaluate indication during dose adjustment review (e.g., new target, no longer indicated)

FOLLOW UP TDM MONITORING

- Recheck tobramycin concentrations ('peak' and 'trough'):
  - Change in tobramycin dose
  - Change in renal function/UOP
  - Addition of nephrotoxic medication, including intravenous contrast (refer to NINJA protocol)
  - Surgical procedure
- Repeat tobramycin concentrations include only <u>one</u> 'random' level if Insight-Rx dosing platform indicates a good model fit ('Fit info' tab shows ✓ next to TDM) at previous TDM

OTHER MONITORING

- Renal function (serum creatinine, BUN, urine output) should be monitored every 24 - 48 hours until stable tobramycin dosing is achieved
- Follow daily serum creatinine per recommendation of NINJA protocol
- Audiology consults should be considered for patients on long-term therapy (≥2 week)
